# Short- and long-term impact of aspirin cessation in older adults: a target trial emulation

**DOI:** 10.1186/s12916-024-03507-8

**Published:** 2024-07-29

**Authors:** Zhen Zhou, Katherine L. Webb, Mark R. Nelson, Robyn L. Woods, Michael E. Ernst, Anne M. Murray, Andrew T. Chan, Andrew Tonkin, Christopher M. Reid, Suzanne G. Orchard, Brenda Kirpach, Raj C. Shah, Nigel Stocks, Jonathan C. Broder, Rory Wolfe

**Affiliations:** 1 School of Public Health and Preventive Medicine, Monash University, Melbourne, VIC Australia; 2 Menzies Institute for Medical Research, University of Tasmania, Hobart, TAS Australia; 3 Department of Pharmacy Practice and Science, College of Pharmacy, The University of Iowa, Iowa, IA USA; 4 Department of Family Medicine, Carver College of Medicine, The University of Iowa, Iowa, IA USA; 5 Division of Geriatrics, Department of Medicine Hennepin HealthCare, Berman Centre for Outcomes and Clinical Research, Hennepin Healthcare Research Institute, Minneapolis, MN USA; 6 University of Minnesota, Minneapolis, MN USA; 7 Clinical and Translational Epidemiology Unit, Massachusetts General Hospital, Boston, MA USA; 8 Division of Gastroenterology, Massachusetts General Hospital, Boston, MA USA; 9 School of Population Health, Curtin University, Perth, WA Australia; 10 Department of Family & Preventive Medicine and the Rush Alzheimer’s Disease Center, Rush University Medical Center, Chicago, IL USA; 11 Discipline of General Practice, University of Adelaide, Adelaide, SA Australia

**Keywords:** Aspirin, Target trial, Cardiovascular disease, Elderly, Cessation, Hemorrhage

## Abstract

**Background:**

The net benefit of aspirin cessation in older adults remains uncertain. This study aimed to use observational data to emulate a randomized trial of aspirin cessation versus continuation in older adults without cardiovascular disease (CVD).

**Methods:**

Post hoc analysis using a target trial emulation framework applied to the immediate post-trial period (2017–2021) of a study of low-dose aspirin initiation in adults aged ≥ 70 years (ASPREE; NCT01038583). Participants from Australia and the USA were included if they were free of CVD at the start of the post-trial intervention period (time zero, *T0*) and had been taking open-label or randomized aspirin immediately before *T0*. The two groups in the target trial were as follows: aspirin cessation (participants who were taking randomized aspirin immediately before *T0*; assumed to have stopped at *T0* as instructed) *versus* aspirin continuation (participants on open-label aspirin at *T0* regardless of their randomized treatment; assumed to have continued at *T0*). The outcomes after *T0* were incident CVD, major adverse cardiovascular events (MACE), all-cause mortality, and major bleeding during 3, 6, and 12 months (short-term) and 48 months (long-term) follow-up. Hazard ratios (HRs) comparing aspirin cessation to continuation were estimated from propensity-score (PS) adjusted Cox proportional-hazards regression models.

**Results:**

We included 6103 CVD-free participants (cessation: 5427, continuation: 676). Over both short- and long-term follow-up, aspirin cessation versus continuation was not associated with elevated risk of CVD, MACE, and all-cause mortality (HRs, at 3 and 48 months respectively, were 1.23 and 0.73 for CVD, 1.11 and 0.84 for MACE, and 0.23 and 0.79 for all-cause mortality, *p* > 0.05), but cessation had a reduced risk of incident major bleeding events (HRs at 3 and 48 months, 0.16 and 0.63, *p* < 0.05). Similar findings were seen for all outcomes at 6 and 12 months, except for a lowered risk of all-cause mortality in the cessation group at 12 months.

**Conclusions:**

Our findings suggest that deprescribing prophylactic aspirin might be safe in healthy older adults with no known CVD.

**Supplementary Information:**

The online version contains supplementary material available at 10.1186/s12916-024-03507-8.

## Background

The net clinical benefit of daily low-dose aspirin initiation in older populations without clinical manifestation of cardiovascular disease (CVD) was unknown until 2018 when the ASPirin in Reducing Events in the Elderly (ASPREE) study was published [1–3]. The ASPREE trial found that aspirin did not extend disability-free survival or reduce CVD events but increased the risk of major bleeding and cancer death. This evidence led to a recommendation in clinical guidelines against aspirin initiation in older adults for primary prevention of CVD [4–6]. Notwithstanding this, a recent US national population survey of 50,000 + respondents showed that 46% of individuals aged ≥ 70 years who had no prior history of CVD reported regular aspirin use in 2019, after the ASPREE trial findings were published [7]. As 89% of participants in the ASPREE trial reported no regular aspirin use prior to enrollment, the main ASPREE results primarily addressed whether aspirin should be initiated in older adults and not whether aspirin should be ceased in those who reach older ages while already taking aspirin [8]. Whether it is safe to cease aspirin remained a subject of debate. A randomized trial of deprescribing aspirin (compared to continuation of aspirin use) would be optimal to provide definitive evidence to answer this question. However, such a trial is unlikely to be conducted, and we recognized that ASPREE participants as they transitioned from the trial to post-trial observational follow-up could be informative.

At the conclusion of the ASPREE trial and after a median of 4.7 years of follow-up, approximately 63% of participants were still taking randomized study medication (62% in the aspirin arm and 64% in the placebo arm) [1–3]. All participants received a notification letter directing them to immediately cease study medication. The letter also advised that any participants taking open-label aspirin by recommendation of their doctor should continue to take their aspirin. This provided a unique opportunity to utilize observational data from the period immediately following the intervention phase of ASPREE to emulate an aspirin deprescribing trial. In this context, we designed an analysis of ASPREE’s post-intervention period to examine whether aspirin cessation, compared to continuation, impacted short- and long-term risk of CVD, all-cause mortality, and major bleeding in participants who had not experienced any CVD-related clinical event and hence had no indication for aspirin use.

## Methods

### Data source

**Fig. 1:**
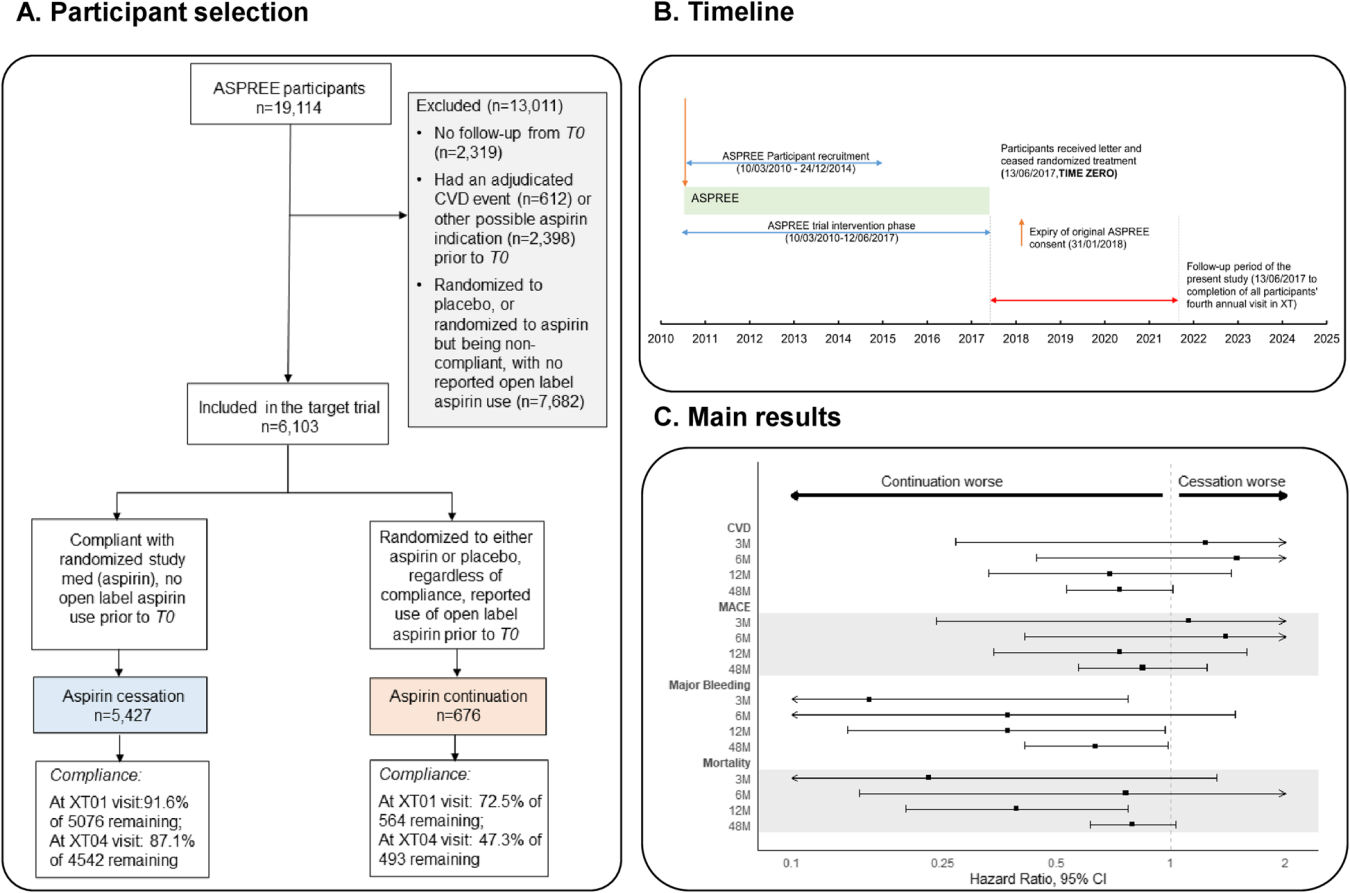
The overall design and main results of this study. **A**, **B** The ASPREE trial intervention phase ended on 12 June 2017, on which day the notification letter was sent to participants to inform the cessation of randomized study medication. Thus, 13 June 2017 serves as the index date (time zero, *T0*) of this analysis. Excluded from this analysis were ASPREE participants who (1) had no continued follow-up after *T0* (*n* = 2319; primary reason was death during the trial) or (2) had adjudicated CVD events (*n* = 612) or reported other possible indications to aspirin use (*n* = 2398; for example, angina or mini stroke) prior to *T0* or (3) had never started aspirin (randomized to placebo and report of no open-label aspirin use before *T0*), which left 6103 participants with whom to emulate a target trial. Among these included participants, those who were originally randomized to aspirin and compliant with the study medication until *T0* (stop) with no report of open-label aspirin use recently prior to *T0* constitute the cessation group (*n* = 5427), and participants who were randomized to either aspirin or placebo and reported recent open-label regular aspirin use prior to *T0* constitute the continuation group (*n* = 676). From *T0*, participants entered an observational phase during which clinical data were collected from participants via scheduled annual in-person visits and 6-monthly phone calls. At the fourth annual XT visit, there remained 47% of participants in the aspirin continuation group reporting the use of open-label aspirin and 87% in the cessation group reporting no aspirin use. **C** The main results showed no increased cardiovascular disease (CVD), major adverse cardiovascular events (MACE), or mortality risks and a lower risk of major bleeding with aspirin cessation. A CVD event was defined as adjudicated non-fatal myocardial infarction, cardiovascular death, stroke, and hospitalization for heart failure. MACE was defined as adjudicated ischemic stroke, myocardial infarction, and coronary heart disease death

We used the post-trial intervention data of ASPREE for this target trial emulation. ASPREE was a large-scale randomized trial (NCT01038583) of low dose aspirin versus placebo among 19,114 adults aged over 70 years (≥ 65 years for US minorities), who were free of cardiovascular events, dementia, and major physical disability at trial enrolment. The recruitment was undertaken between 2010 and 2014, and the participants were followed up for a median of 4.7 years. The intervention phase of the ASPREE trial ended on 12 June 2017 when the notification letter was sent to all participants. Study visits continued to January 2018 without intervention. From 13 June 2017, participants provided consent to ASPREE-eXTension (ASPREE-XT), an extended observational period [9]. The occurrence of health events after 12 June 2017 continued to be followed up with the same rigor as during the trial intervention phase. After the trial intervention phase, participants remained blinded to their study medication assignment and unaware of the principal findings of the trial for a further 15 months. An unblinding letter was sent to all participants on 14 September 2018 when the main findings of the ASPREE trial were published. The timeline of the ASPREE trial intervention phase and post-intervention phase for our analysis is presented in Fig. 1.

### Study design

We adopted a target trial framework to guide our analysis using observational data from ASPREE-XT that would emulate a randomized trial comparing aspirin cessation to continuation [10–12]. The emulation involved two main steps. The first step was to clearly define the causal question within a hypothetical randomized trial protocol, which included the defining of eligibility criteria, treatment strategies, treatment assignment, start (time zero, henceforth referred to as “*T0*”) and end of follow-up, outcomes, causal contrasts, and planned statistical analysis. The second step was to emulate these trial protocol components using the observational data. Additional file 1: Table S1 outlines the detailed specifications of the target trial emulated for this study.

### Eligibility criteria

The original selection criteria of the ASPREE trial applied [1–3]. Additionally, only surviving individuals at *T0* were included in the target trial and only if at *T0* they had remained free of any adjudicated cardiovascular event and did not have a reported likely cardiovascular event or use of medication for likely secondary prevention purpose. In other words, an exclusion criterion was any possible clinical indication for aspirin. Adjudicated cardiovascular events included non-fatal myocardial infarction (MI), coronary heart disease death, stroke, and hospitalization for heart failure (HF), all of which were adjudicated by an expert clinical panel. Cardiovascular events self-reported or recorded in medical reports included events sent to the MI, HF, or stroke adjudication committee but rejected as not meeting the outcome criteria, and angina, heart/chest pain, transient ischemic attack, atrial fibrillation, deep vein thrombosis/pulmonary embolism, or any cardiac/vascular hospitalization and self-report of medications commonly used for secondary prevention included nitrates, ranolazine, or platelet aggregation inhibitors excluding aspirin.

### Start and end of follow-up

*T0* (13 June 2017) was defined as the day following the dispatch of the letter notifying the end of trial intervention. All participants were followed up until the incidence of outcome events, death, or their fourth ASPREE-XT annual visit (between 2021 and 2022), whichever occurred first (Fig. 1). During both ASPREE and ASPREE-XT periods, clinical data were collected from participants via scheduled annual in-person visits and 6-monthly phone calls. For those who were unable to be contacted, their medical records were reviewed. Data of aspirin use and other concomitant medications were gathered either from primary care physician records by research staff or through self-reports of current medication use during annual visits. Compliance to randomized aspirin was determined by count of used pills > 0 from returned study medication bottles, and compliance to open-label aspirin was based on self-reported use at each annual visit.

### Treatment strategies and assignments

We compared participants who continued taking aspirin versus those who discontinued it. The continuation group consisted of participants who reported recent open-label aspirin use at their most recent annual visit before *T0* (average 184 days prior to *T0*), regardless of their ASPREE randomized assignment. We assumed that participants in the continuation group were still taking aspirin at *T0* and continued doing so thereafter. The cessation group consisted of participants from the randomized aspirin arm, who, based on a count of returned study pills, were determined to have taken randomized aspirin in the last calendar year of trial intervention (year 2017) and did not report recent open-label aspirin use before *T0*. We assumed that participants in the cessation group were still taking randomized aspirin at the time of receiving the notification letter and immediately ceased use as instructed. These assumptions of adherence to the course of action instructed in the letter emulate an intention-to-treat analysis approach in a randomized trial [11]. Treatment randomization was emulated through a propensity score (PS)-based method, as detailed in the “Statistical analysis” section.

### Outcomes

Study outcomes were incident CVD (non-fatal MI, cardiovascular death, stroke, and hospitalization for HF), major adverse cardiovascular events (MACE), all-cause mortality, and major bleeding events. MACE included fatal and non-fatal ischemic stroke, non-fatal MI, and coronary heart disease death. Major bleeding events included clinically significant bleeding, hemorrhagic stroke, and subarachnoid hemorrhage. Clinically significant bleeding was defined as bleeding that led to transfusion, hospitalization, prolongation of hospitalization, surgery, or death. For analysis of major bleeding, participants who had major bleeding events before *T0* were further excluded. All clinical events in these outcomes were adjudicated by panels of clinical experts who were masked to treatment randomization and follow-up status with respect to aspirin use [1–3].

### Causal contrast

Using an intention-to-treat approach, this study compared aspirin cessation versus continuation at *T0*, regardless of subsequent treatment crossovers during the follow-up period.

### Statistical analysis

To emulate random treatment assignment, we created a propensity score (PS) of aspirin cessation for each participant using logistic regression, with the following covariates: age, gender, race/ethnicity/country (white Australian, white US, Hispanic, black, other), systolic and diastolic blood pressure, total cholesterol (TC), triglycerides, estimated glomerular filtration rate (eGFR using CKD-EPI equation [13]), smoking (current/former vs. never), diabetes, education (< 12 vs. ≥ 12 years), use of statins, anti-hypertensives, non-steroidal anti-inflammatory drugs (NSAIDs), and antithrombotic agents (excluding aspirin), and family history of MI. Covariate values came from annual visits that occurred within 2 years prior to *T0*; if unavailable from these visits, values were imputed using a linear model (< 0.5% of covariate values). Conceptually, the PS was equivalent to one minus the propensity of being on open-label aspirin for primary prevention at *T0*. Differences in covariates between the two groups before and after applying the PS approach were checked by using an absolute standardized difference (ASD) measure, where a difference ≥ 0.1 indicates imbalance.

For outcome analysis, Cox proportional-hazards regression models were used to generate hazard ratios (HRs) and 95% confidence intervals (CIs) of each outcome at 3, 6, and 12 months and over the full time period of follow-up (approximately 4 years) after *T0*, with adjustment made for the PS [14]. The proportional hazards assumption was assessed with Schoenfeld residuals, and no violations were found.

Three sensitivity analyses were performed with different eligibility criteria: (1) inclusion of participants who had a possible aspirin indication, to assess the impact of aspirin cessation in all participants without confirmed usage for secondary prevention (2) excluding participants who reported the use of other antithrombotic medications for primary prevention which might increase the risk of bleeding associated with aspirin; and (3) including more covariates in the propensity score that are associated with CVD and death risk in older people, including high-density-lipoprotein cholesterol (HDL-c), fasting plasma glucose, alcohol consumption, body mass index (BMI), grip strength, and gait speed.

We additionally performed a per-protocol analysis using an inverse-probability-of-censoring weights (IPCW) approach [15]. Endpoint-specific analyses were conducted in which participants remaining alive in primary prevention were right-censored at the first occurrence of continuation/cessation nonadherence. We applied IPCW to upweight remaining participants for imparted informative censoring at selected time points. Non-adherence was defined in the cessation group as the initiation of open-label aspirin; in the continuation group, it was the cessation of open-label aspirin. Weights were calculated separately for the two treatment groups using the same set of covariates used for the propensity score and additionally time-varying covariates for CVD events (in mortality and major hemorrhage analyses) and major hemorrhage events (in CVD, MACE, and mortality analyses). The product of time-varying IPCW and time-fixed PS weights were used in weighted Cox proportional hazards models [16].

All statistical tests were 2-sided, and *p* < 0.05 was considered statistically significant. Analyses were conducted using R version 4.0.2 (R Core Team, 2020).

### Role of the funding source

The funding sources and drug provider played no role in the design, conduct, and reporting of the ASPREE trial, ASPREE-XT, and this analysis.

## Results

### Study population and baseline characteristics

A flow chart of participant selection is presented in Fig. 1 and Additional file 1: Fig. S1. After excluding 2319 participants with no follow-up beyond *T0*, 3010 with an adjudicated cardiovascular event and/or other possible aspirin indications, and 7682 who did not take aspirin or lacked relevant information to confirm their aspirin use before *T0*, we included 6103 participants for the final analysis, with 676 (11.0%) categorized into the aspirin continuation group and 5427 (89.0%) into the cessation group. Adherence to aspirin use at > 1 year was 72.5% in the continuation group, and adherence to aspirin non-use was 91.6% in the cessation group at this time point. Adherence rates remained high at approximately 4.5 years after *T0* (Fig. 1). Only 47 (8 [1.2%] continuation, 39 [0.7%] cessation) participants withdrew during follow-up, with a mean (SD) follow-up time of 1.9 (1.2) years.

Compared with the aspirin continuation group, participants in the aspirin cessation group were, on average, slightly younger and more likely to be Australian and had higher total cholesterol and eGFR levels. The cessation group also had a lower prevalence of self-reported diabetes and family history of MI and was less likely to use statins, anti-hypertensive agents, and antithrombotic medications. Participants in the continuation group had a higher prevalence of self-reported aspirin use prior to ASPREE, although this variable was not adjusted for in the PS model. After applying PS, all covariates were well-balanced (ASDs < 0.1) (Table 1).

**Table 1:** Participant characteristics at *time zero* (13 June 2017)

	**Overall (** ***n*** ** = 6103)**	**Aspirin continuation (** ***n*** ** = 676)**	**Aspirin cessation (** ***n*** ** = 5427)**	**Absolute standardized difference (ASD)**	
				**Before**	**After**
Age, years (min–max)	79.2 ± 4.4 (67.9–100.2)	80.0 ± 4.8 (68.0–97.1)	79.1 ± 4.3 (67.9–100.2)	0.21	0.05
Male gender	2661 (43.6)	292 (43.2)	2369 (43.7)	< 0.01	< 0.01
Race/ethnicity/country					
White Australian	5293 (86.7)	504 (74.6)	4789 (88.2)	0.14	< 0.01
White US	339 (5.6)	72 (10.7)	267 (4.9)	0.06	< 0.01
Hispanic	253 (4.1)	59 (8.7)	194 (3.6)	0.05	< 0.01
Black	135 (2.2)	28 (4.1)	107 (2.0)	0.02	< 0.01
Other	83 (1.4)	13 (1.9)	70 (1.3)	< 0.01	< 0.01
Education ≥ 12 years	3448 (56.5)	414 (61.2)	3034 (55.9)	0.05	< 0.01
Total cholesterol, mg/DL	197.2 ± 38.8	188.3 ± 41.6	198.3 ± 38.3	0.26	0.02
Triglycerides, mg/DL	118.5 ± 56.6	117.7 ± 53.6	118.6 ± 56.9	0.02	0.03
SBP, mmHg	136.9 ± 16.9	137.1 ± 17.3	136.9 ± 16.8	0.01	0.01
DBP, mmHg	74.2 ± 9.7	74.0 ± 9.8	74.2 ± 9.7	0.02	0.01
Smoking, ever	2621 (42.9)	302 (44.7)	2319 (42.7)	0.02	< 0.01
eGFR, ml/min/1.73 m^2^	69.9 ± 14.4	68.8 ± 15.4	70.0 ± 14.2	0.09	< 0.01
Self-report diabetes	600 (9.8)	98 (14.5)	502 (9.3)	0.05	< 0.01
Statin use	2206 (36.1)	341 (50.4)	1865 (34.4)	0.16	0.02
Anti-hypertensive agents use	3630 (59.5)	475 (70.3)	3155 (58.1)	0.12	0.02
NSAID use	901 (14.8)	117 (17.3)	784 (14.4)	0.03	< 0.01
Anti-thrombotic use (excluding aspirin)	466 (7.6)	92 (13.6)	374 (6.9)	0.07	< 0.01
Family history of MI	2571 (42.1)	320 (47.3)	2251 (41.5)	0.06	< 0.01
Pre-trial aspirin use (*not adjusted*)	675 (11.1)	134 (19.8)	541 (10.0)	–	–

*Abbreviations*: *SBP* systolic blood pressure, *DBP* diastolic blood pressure, *eGFR* estimated glomerular filtration rate, *MI* myocardial infarction, *NSAIDs* non-steroidal anti-inflammatory drugs

Continuous variables are presented as mean ± SD and categorical variables are presented as *n* (%)

### Short-term follow-up (3–12 months)

**Fig. 2:**
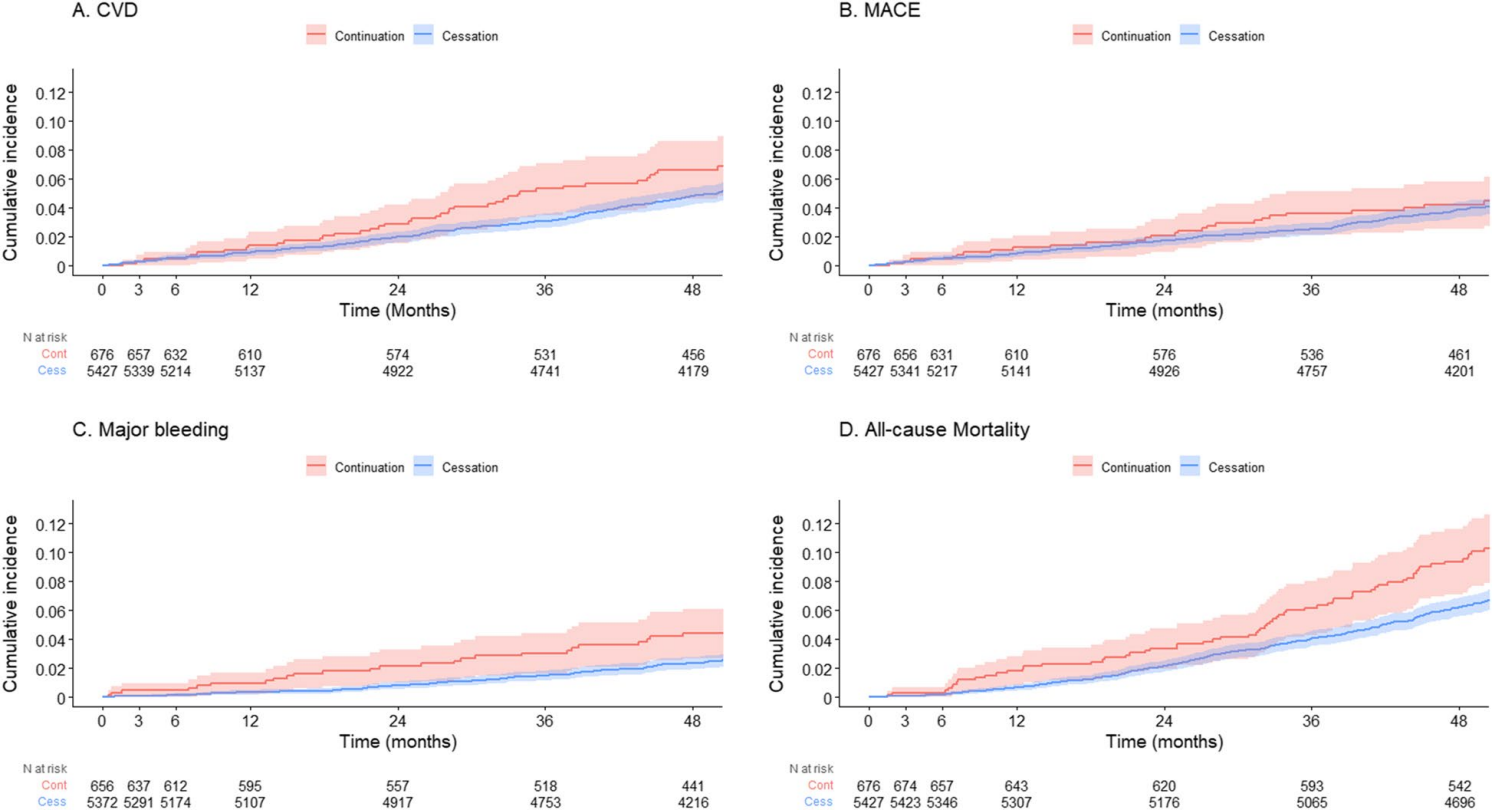
Cumulative incidence plots for CVD (**A**), MACE (**B**), major bleeding (**C**), and all-cause mortality (**D**) in the aspirin continuation and cessation groups. The red line represents the continuation group (Cont), and the blue line represents the cessation group (Cess). The shading denotes 95% confidence intervals. Abbreviations: CVD, cardiovascular disease; MACE, major adverse cardiovascular event

The observed incidence rates of CVD and MACE outcomes were comparable between the cessation and continuation groups at 3 and 6 months and were numerically lower in the cessation group at 12 months, while the observed incidence rates of all-cause mortality and major bleeding events were numerically lower in the cessation group than the continuation group throughout the follow-up period (Table 2). The PS-adjusted models found no statistically significant association between aspirin cessation and incident CVD events at 3, 6, and 12 months (PS-adjusted HRs [95% CI]: 1.23 [0.27–5.58] at 3 months, 1.49 [0.44–5.03] at 6 months, and 0.69 [0.33–1.44] at 12 months, all *p* values ≥ 0.30); also, there was no significant association between aspirin cessation and MACE (PS-adjusted HRs [95% CI]: 1.11 [0.24–5.13] at 3 months, 1.39 [0.41–4.73] at 6 months, and 0.73 [0.34–1.58] at 12 months, all *p* values ≥ 0.40). Aspirin cessation was associated with a significantly lower risk of all-cause mortality at 12 months (PS-adjusted HR [95% CI]: 0.39 [0.20–0.77]) but not at 3 and 6 months (0.23 [0.04–1.32] at 3 months and 0.76 [0.15–3.75] at 6 months). For bleeding, cessation of aspirin was associated with an 84% (PS-adjusted HR [95% CI]: 0.16 [0.03–0.77]), 63% (0.37 [0.09–1.47]), and 63% (0.37 [0.14–0.96]) reduced risk of incident major bleeding events at 3, 6, and 12 months respectively (Fig. 1, Table 2), with the association being statistically significant at 3 and 12 months. Cumulative incidence curves of major bleeding events for the two groups diverged from *T0*, indicating that bleeding risk lowered soon after discontinuing aspirin (Fig. 2).

**Table 2:** The hazard of each study outcome in the aspirin cessation group versus the continuation group during four follow-up periods

	**Events**	**Incidence rate (per 1000 py)**	**Unadjusted HR (95% CI)**	***p***	**PS-adjusted HR (95% CI)**	***p***
**CVD (*n *= 6103)**						
3 months						
Continuation	2	11.85	Ref	1.00	Ref	0.79
Cessation	16	11.81	1.00 (0.23, 4.34)		1.23 (0.27, 5.58)	
6 months						
Continuation	3	8.90	Ref	0.72	Ref	0.52
Cessation	30	11.09	1.25 (0.38, 4.08)		1.49 (0.44, 5.03)	
12 months						
Continuation	9	13.39	Ref	0.28	Ref	0.32
Cessation	49	9.07	0.68 (0.33, 1.38)		0.69 (0.33, 1.44)	
48 months						
Continuation	46	18.20	Ref	0.03	Ref	0.06
Cessation	281	12.94	0.71 (0.52, 0.96)		0.73 (0.53, 1.01)	
**MACE (*n* = 6103)**						
3 months						
Continuation	2	11.85	Ref	0.86	Ref	0.89
Cessation	14	10.33	0.87 (0.20, 3.84)		1.11 (0.24, 5.13)	
6 months						
Continuation	3	8.90	Ref	0.85	Ref	0.60
Cessation	27	9.98	1.12 (0.34, 3.70)		1.39 (0.41, 4.73)	
12 months						
Continuation	8	11.90	Ref	0.35	Ref	0.42
Cessation	45	8.33	0.70 (0.33, 1.48)		0.73 (0.34, 1.58)	
48 months						
Continuation	31	12.22	Ref	0.33	Ref	0.38
Cessation	221	10.16	0.83 (0.57, 1.21)		0.84 (0.57, 1.24)	
**Major bleeding**^a^ **(*n*** **= 6028)**						
3 months						
Continuation	3	18.35	Ref	0.02	Ref	0.02
Cessation	4	2.98	0.16 (0.04, 0.73)		0.16 (0.03, 0.77)	
6 months						
Continuation	3	9.18	Ref	0.10	Ref	0.16
Cessation	8	2.98	0.32 (0.09, 1.22)		0.37 (0.09, 1.47)	
12 months						
Continuation	6	9.20	Ref	0.02	Ref	0.04
Cessation	17	3.17	0.34 (0.14, 0.87)		0.37 (0.14, 0.96)	
48 months						
Continuation	26	10.59	Ref	0.01	Ref	0.04
Cessation	136	6.27	0.58 (0.38,0.89)		0.63 (0.41, 0.98)	
**All-cause mortality (*n* = 6103)**						
3 months						
Continuation	2	11.85	Ref	0.11	Ref	0.10
Cessation	4	2.95	0.25 (0.05, 1.36)		0.23 (0.04, 1.32)	
6 months						
Continuation	2	5.93	Ref	0.46	Ref	0.73
Cessation	9	3.32	0.56 (0.12, 2.59)		0.76 (0.15, 3.75)	
12 months						
Continuation	12	17.88	Ref	< 0.01	Ref	0.01
Cessation	35	6.47	0.36 (0.19, 0.70)		0.39 (0.20, 0.77)	
48 months						
Continuation	69	25.06	Ref	< 0.01	Ref	0.08
Cessation	395	17.18	0.68 (0.53, 0.88)		0.79 (0.61, 1.03)	

*Abbreviations*: *CVD* cardiovascular disease, *MACE* major adverse cardiovascular events, *PS* propensity score, *HR* hazard ratio, *CI* confidence interval

^a^A total of 75 participants who experienced major bleeding prior to 13 June 2017 were removed from the major bleeding analysis

### Long-term follow-up (48 months)

Compared to the aspirin continuation group, the cessation group had a lower incidence rate of CVD and MACE over long-term follow-up (CVD: 12.9 versus 18.2 events per 1000 person-years; MACE: 10.2 versus 12.2 events per 1000 person-years). No significant association with aspirin cessation was found for CVD, MACE, and all-cause mortality (CVD, PS-adjusted HR [95% CI]: 0.73 [0.53–1.01], *p* = 0.06; MACE: PS-adjusted HR [95% CI]: 0.84 [0.57–1.24], *p* = 0.38; all-cause mortality, PS-adjusted HR [95% CI]: 0.79 [0.61–1.03], *p* = 0.08). For major bleeding, there was a similar finding during the extended follow-up as the short-term follow-up period with a 37% reduced risk of bleeding with aspirin cessation (PS-adjusted HR [95% CI]: 0.63 [0.41–0.98], *p* = 0.04). (Table 2, Fig. 1 and Fig. 2).

Congruent with the intention-to-treat analysis, the per-protocol analysis found no significant associations of aspirin cessation with CVD, MACE, and all-cause mortality at 48 months and a significant association between aspirin cessation and a reduced risk of major bleeding (IPCW- and PS-adjusted HR [95% CI]: 0.48 [0.29, 0.79]) at 48 months (Table 3). Results from other sensitivity analyses with altered eligibility criteria were also generally congruent with the main short-term and long-term findings, except for the non-significant (*p* = 0.06) reduction of major bleeding at 48 months in the analysis including more covariates in the PS model (Additional file 1: Tables S2-S5).

**Table 3:** Comparison of intention-to-treat (PS-adjusted) and per-protocol (IPCW- and PS-adjusted) hazard ratios of each study outcome in the aspirin cessation versus continuation group during long-term follow-up to 48 months

	**PS-adjusted HR (95% CI)**	***p***	**IPCW- and PS-adjusted HR (95% CI)**	***p***
**CVD (*n* = 6103)**				
48 months				
Continuation	Ref	0.06	Ref	0.30
Cessation	0.73 (0.53, 1.01)		0.80 (0.53, 1.22)	
**MACE (*n* = 6103)**				
48 months				
Continuation	Ref	0.38	Ref	0.65
Cessation	0.84 (0.57, 1.24)		0.89 (0.55, 1.46)	
**Major bleeding**^a^ **(*n *= 6028)**				
48 months				
Continuation	Ref	0.04	Ref	0.004
Cessation	0.63 (0.41, 0.98)		0.48 (0.29, 0.79)	
**All-cause mortality (*n*** **= 6103)**				
48 months				
Continuation	Ref	0.08	Ref	0.14
Cessation	0.79 (0.61, 1.03)		0.77 (0.54, 1.09)	

*Abbreviations*: *CVD* cardiovascular disease, *MACE* major adverse cardiovascular events, *PS* propensity score, *IPCW* inverse probability of censoring weight, *HR* hazard ratio, *CI* confidence interval

^a^A total of 75 participants who experienced major bleeding prior to 13 June 2017 were removed from the major bleeding analysis

## Discussion

Compared with aspirin continuation, there was no evidence of an increase in the rates of incident CVD or MACE events or all-cause mortality over both short-term (3–12 months) and long-term follow-up (48 months) periods among participants who ceased aspirin at the end of the trial. However, aspirin cessation was associated with a significantly reduced risk of major bleeding by between 63 and 84% when compared to aspirin continuation during the 3- to 12-month timeframe and by 37% during the extended follow-up. Our findings support the safety of stopping aspirin in those aged ≥ 70 years where it is being used in the context of primary CVD prevention.

The safety of discontinuing daily preventive aspirin has been a great concern due to a proposed risk of unmasking of subclinical CVD and prothrombotic rebound effects that could prompt a CVD event [17, 18]. Aspirin discontinuation leads to an inevitable progressive recovery of cyclooxygenase-1 (COX-1) activity that promotes the production of prostaglandin, which is converted to thromboxane-A_2_ (TXA_2_) by thromboxane synthase in fresh platelets [19]. TXA_2_ promotes platelet activation and aggregation on the surface of the damaged vessel walls leading to a thrombotic occlusion and subsequently an acute thrombotic event [20, 21]. It has been estimated that acute coronary artery and cerebrovascular events could be triggered within 7 days to 4 weeks following aspirin cessation [19–22]. However, in this study, we found no noticeable increase in risks of CVD and MACE with aspirin cessation at 3-month follow-up. In addition, there was no significant association between aspirin cessation, CVD, and MACE over a longer follow-up (6, 12, and 48 months). These insights support the hypothesis that ceasing aspirin reduces clinically significant bleeding with no rebound effect linked to increased CVD risk. In line with our findings, a randomized trial comparing the short-term effect of continued aspirin versus placebo in 4382 patients undergoing noncardiac surgery who had been taking aspirin for vascular conditions before trial entry found no evidence of an immediate aspirin withdrawal effect [23]. In that study, aspirin continuation was not associated with the risk of a composite outcome of death or non-fatal myocardial infarction (HR [95% CI]: 1.00 [0.81–1.24]) at 30 days, but an increased risk of the composite outcome of life-threatening and major bleeding was observed although this finding was not statistically significant (HR [95% CI]: 1.20 [0.94–1.55]).

The significant decrease in major bleeding risk with aspirin cessation throughout the entire follow-up period and a decreased risk in all-cause mortality with aspirin cessation at 12 months in our analysis supports clinical guidelines that recommend against aspirin use in this age group for primary prevention due to its significantly increased risk of major bleeding events and lack of overall benefit [4–6]. The weakened association between aspirin continuation and bleeding risk at 4-year follow-up may be explained by the participants on open-label aspirin being made aware of the ASPREE trial results at 15 months after *T0* and opting to stop open-label aspirin use at that time. When analyses were adjusted for treatment adherence, the evidence of elevated bleeding risk in the continuation group was even more compelling. The cumulative incidence plot suggests that the bleeding risk between the aspirin cessation and continuation groups differentiated soon after the commencement of follow-up. These findings agree with our previous analysis of the ASPREE participants when they entered the trial, approximately 4–5 years earlier [24]. In that post hoc analysis, we restricted to individuals reporting aspirin use prior to randomization (≥ 2 days/week) and investigated the effect of aspirin cessation (randomized placebo) versus continuation (randomized aspirin) at the start of trial. Discontinuing aspirin (through randomization to placebo) had no impact on CVD, MACE, all-cause mortality, and major bleeding events compared with continued aspirin use over a median of 4.7-year follow-up [24]. The robustness of that analysis was limited by the small sample size (*n* = 1714) and a 4-week placebo run-in phase for the trial that prevented capture of the short-term unmasking effect, although the findings were echoed in meta-analysis with another similar trial [25]. The present study reported here reinforces our conclusions from the previous analysis, and together the findings reassure the benefits of aspirin cessation (reducing bleeding risk) in older adults in the context of primary CVD prevention, an aspect which has often been less emphasized compared with its potential in increasing CVD risk. More studies with high-quality data and larger sample sizes are warranted to confirm our results [26].

This study has limitations. First, we assumed that all participants on randomized aspirin before *T0* would immediately stop taking the study medication after receiving the letter, and those who reported open-label aspirin use in the most recent follow-up visit before *T0* would continue aspirin thereafter. These assumptions could lead to misclassification bias, likely towards null effects; however, the data we presented on aspirin use during follow-up indicated that the assumptions were reasonable. Furthermore, a per-protocol analysis was performed so as not to underestimate safety concerns of deprescribing aspirin; results from these were consistent with our main findings for the long-term effects, further indicating that our assumptions were reasonable. Second, the event numbers in the relatively short timeframe of follow-up were small for all outcomes, leading to wide CIs for the 3-, 6-, and 12-month effect estimates. The data presented do not out rule the possibility of an adverse effect of aspirin cessation on CVD at 6 months. Third, our findings were based on a sample constituting predominantly white healthy older adults and may not be generalizable to other race/ethnicity groups or populations in different settings. Last, the aspirin continuation group seemed to have a higher CVD risk at *T0*, despite the exclusion of participants with possible and probable CVD events or using of medication for likely secondary prevention purpose. Although the propensity-score approach was employed to account for the higher CVD risk and minimize potential confounding, the presence of residual bias in comparing aspirin cessation with continuation cannot be ruled out.

Notwithstanding these limitations, this analysis has some key strengths including using a robust approach to emulate a randomized trial via the PS technique, the use of well-characterized, highly complete data of older individuals with rigorous long-term follow-up, adjudication of all study endpoints, and the ability to identify all participants with a possible aspirin indication at time zero.

## Conclusions

This analysis of observational data to emulate a deprescribing trial found no short-term or long-term harmful impact of aspirin cessation on the risk of CVD and all-cause mortality and a reduced risk of major bleeding events. These findings suggest that deprescribing aspirin might be safe in healthy individuals with no known CVD who are aged 70 years or above.

### Supplementary Information

Additional file 1: Fig. S1. Participant selection flowchart. Table S1. Target trial framework. Table S2. Outcome analysis additionally including participants with possible aspirin indications. Table S3. Outcome analysis additionally excluding participants with antithrombotic use. Table S4. Participant characteristics at T0 with additional variables included. Table S5. Outcome analysis including additional covariates.

## Data Availability

Requests for data access will be via the ASPREE principal investigators with details for applications provided through https://aspree.org/aus/for-researchers/ or https://aspree.org/usa/for-researchers/. The DOI of ASPREE is: 10.26180/21715979.v2.

## Abbreviations

ASD: Absolute standardized difference
ASPREE: Aspirin in Reducing Events in the Elderly
ASPREE-XT: ASPREE-eXTension
BMI: Body mass index
CI: Confidence interval
CKD-EPI: Chronic Kidney Disease Epidemiology Collaboration
COX-1: Cyclooxygenase-1
CVD: Cardiovascular disease
eGFR: Estimated glomerular filtration rate
HDL-C: High-density lipoprotein cholesterol
HF: Heart failure
HR: Hazard ratio
IPCW: Inverse probability of censoring weighting
MACE: Major adverse cardiovascular events
MI: Myocardial infarction
NSAID: Non-steroidal anti-inflammatory drug
PS: Propensity score
TC: Total cholesterol
TXA2: Thromboxane-A2
