# Supplementary material for: Short- and long-term impact of aspirin cessation in older adults: a target trial emulation
Source: BMC Med. 2024 Jul 29;22:306. doi: 10.1186/s12916-024-03507-8 (PMC11287830; doi:10.1186/s12916-024-03507-8)
Supplement: Supplementary file 1 — Additional file 1: Fig. S1. Participant selection flowchart. Table S1. Target trial framework. Table S2. Outcome analysis additionally including participants with possible aspirin indications. Table S3. Outcome analysis additionally excluding participants with antithrombotic use. Table S4. Participant characteristics at T0 with additional variables included. Table S5. Outcome analysis including additional covariates. [file 12916_2024_3507_MOESM1_ESM.docx]

**Fig S1.** Participant selection flowchart.


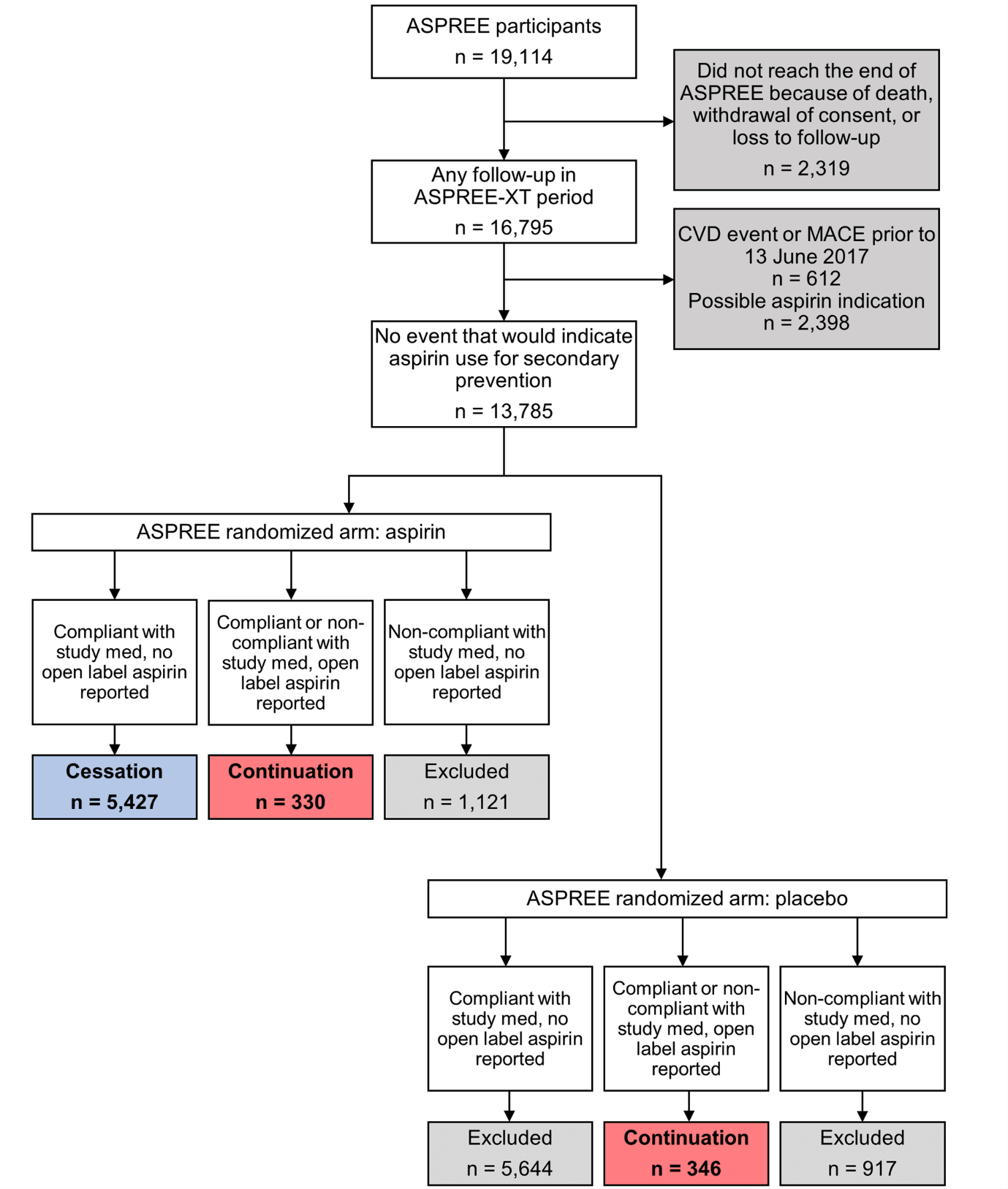


Study medication compliance and open-label aspirin report were collected at the annual visit in the year prior to 13 June 2017. Compliance was determined by count of used pills >0 from returned study medication bottles.

Cardiovascular disease (CVD) event was defined as adjudicated non-fatal myocardial infarction, cardiovascular death, stroke, hospitalization for heart failure. Major adverse cardiovascular event (MACE) was defined as adjudicated ischemic stroke, myocardial infraction (MI), and coronary heart disease death. Possible aspirin indication was defined as self-report of angina, heart/chest pain, transient ischemic attack, atrial fibrillation, deep vein thrombosis/ pulmonary embolism, any cardiac/vascular hospitalization; self-report of medications commonly used for secondary prevention (nitrates, ranolazine, or platelet aggregation inhibitors excluding aspirin); or any event sent to the MI, heart failure, or stroke adjudication committee but rejected as not meeting the outcome criteria.^4-6^

**Table S1.** Target trial framework.

| **Characteristics** | **Target trial** |
| --- | --- |
| **Aim** | To investigate the effect of cessation of primary prevention aspirin use on the short-term and long-term risks of incident cardiovascular events and major bleeding events. |
| **Data** | ASPREE trial & ASPREE-XT (post-trial extended observational period). |
| **Eligibility** | Inclusion criteria:   - All ASPREE inclusion criteria. ^4-6^ - Available data during ASPREE trial on possible aspirin indications. - Available follow-up data during ASPREE-XT.   Exclusion criteria:   - Having an adjudicated CVD event before 13 June 2017. - Died before 13 June 2017. - Assigned to the randomized placebo group and with no reported open-label aspirin use prior to 13 June 2017. - Possible aspirin indication prior to 13 June 2017, defined as self-report of angina, heart/chest pain, transient ischemic attack, atrial fibrillation, deep vein thrombosis/ pulmonary embolism, any cardiac/vascular hospitalization; self-report of medications commonly used for secondary prevention (nitrates, ranolazine, or any antithrombotic agents excluding aspirin); or any event sent to the MI, heart failure, or stroke adjudication committee but adjudicated as a non-outcome. |
| **Follow-up** | Follow-up begins from 13 June 2017 (the time when notification letter was assumed to be received by participants, “***TIME ZERO, T0***”).  Timeframes of follow-up: 3, 6, and 12 months (short-term) and 4 years (long-term). |
| **Treatment strategies** | **Aspirin cessation:** participants who were originally randomized to aspirin and continued to take their study medication until time zero (*T0*) when they received the notification letter directing them to cease.  **Aspirin continuation:** participants (randomized to either aspirin or placebo) who reported recent open-label aspirin use prior to *T0* (no requirement made on exposure time and frequency). |
| **Treatment assignment** | Randomization emulated through a propensity-score (PS) based method. |
| **Outcomes** | - **CVD:** cardiovascular death, non-fatal MI, fatal and non-fatal stroke, any hospitalization for heart failure. - **MACE:** Ischemic stroke, non-fatal MI, coronary heart disease (CHD) death. - **Major bleeding:** Clinically significant bleeding, hemorrhagic stroke, or subarachnoid hemorrhage. - **All-cause mortality.** |
| **Causal contrast** | Aspirin cessation versus continuation (according to assumptions about allocation to treatment strategy on *T0*), regardless of treatment adherence and crossovers during the follow-up of this analysis. |
| **Statistical analysis** | Cox proportional hazards models adjusted for PS. |
| **Limitations** | - Assumes that open-label aspirin reported at a single visit prior to *T0* confirms open-label aspirin usage. - Assumes that participants who started taking open-label aspirin during the trial period continued taking their open-label aspirin as advised in the notification letter they received from ASPREE on *T0*. - Assumes that participants in the aspirin cessation group ceased study medication immediately when they received the notification letter advising them to cease study medication and did not start open-label aspirin after *T0.* |

**Table S2.** The hazard of each study outcome in the aspirin cessation versus continuation group during four follow-up periods (additionally including participants with possible aspirin indications**).

|  | **Events** | **Incidence Rate (per 1000py)** | **Unadjusted HR**  **(95% CI)** | **p** | **PS-adjusted HR (95% CI)** | **p** |
| --- | --- | --- | --- | --- | --- | --- |
| **CVD (n = 7,341)** | |  |  |  |  |  |
| 3 months |  |  |  |  |  |  |
| Continuation | 5 | 13.31 | Ref. | 0.80 | Ref. | 0.94 |
| Cessation | 17 | 11.67 | 0.88 (0.32, 2.38) |  | 0.96 (0.29, 3.21) |  |
| 6 months |  |  |  |  |  |  |
| Continuation | 10 | 13.33 | Ref. | 0.72 | Ref. | 0.54 |
| Cessation | 34 | 11.69 | 0.88 (0.43, 1.78) |  | 1.31 (0.55, 3.12) |  |
| 12 months |  |  |  |  |  |  |
| Continuation | 22 | 14.71 | Ref. | 0.11 | Ref. | 0.77 |
| Cessation | 57 | 9.82 | 0.67 (0.41, 1.09) |  | 0.91 (0.49, 1.69) |  |
| 48 months |  |  |  |  |  |  |
| Continuation | 105 | 18.45 | Ref. | <0.01 | Ref. | 0.25 |
| Cessation | 312 | 13.40 | 0.72 (0.58, 0.90) |  | 0.85 (0.65, 1.12) |  |
| **MACE (n = 7,341)** | |  |  |  |  |  |
| 3 months |  |  |  |  |  |  |
| Continuation | 4 | 10.64 | Ref. | 0.95 | Ref. | 0.99 |
| Cessation | 15 | 10.3 | 0.97 (0.32, 2.92) |  | 1.01 (0.27, 3.81) |  |
| 6 months |  |  |  |  |  |  |
| Continuation | 8 | 10.66 | Ref. | 0.87 | Ref. | 0.78 |
| Cessation | 29 | 9.97 | 0.94 (0.43, 2.05) |  | 1.14 (0.44, 2.97) |  |
| 12 months |  |  |  |  |  |  |
| Continuation | 18 | 12.02 | Ref. | 0.20 | Ref. | 0.43 |
| Cessation | 49 | 8.44 | 0.70 (0.41, 1.2) |  | 0.76 (0.39, 1.48) |  |
| 48 months |  |  |  |  |  |  |
| Continuation | 71 | 12.44 | Ref. | 0.14 | Ref. | 0.41 |
| Cessation | 239 | 10.24 | 0.82 (0.63, 1.07) |  | 0.87 (0.63, 1.21) |  |
| **Major bleeding* (n = 7,216)** | | |  |  |  |  |
| 3 months |  |  |  |  |  |  |
| Continuation | 9 | 24.83 | Ref. | <0.01 | Ref. | 0.03 |
| Cessation | 5 | 3.47 | 0.14 (0.05, 0.42) |  | 0.23 (0.06, 0.88) |  |
| 6 months |  |  |  |  |  |  |
| Continuation | 12 | 16.58 | Ref. | <0.01 | Ref. | 0.03 |
| Cessation | 9 | 3.13 | 0.19 (0.08, 0.45) |  | 0.31 (0.10, 0.92) |  |
| 12 months |  |  |  |  |  |  |
| Continuation | 19 | 13.15 | Ref. | <0.01 | Ref. | 0.01 |
| Cessation | 19 | 3.30 | 0.25 (0.13, 0.47) |  | 0.34 (0.15, 0.75) |  |
| 48 months |  |  |  |  |  |  |
| Continuation | 75 | 13.64 | Ref. | <0.01 | Ref. | 0.05 |
| Cessation | 154 | 6.64 | 0.48 (0.37, 0.64) |  | 0.70 (0.49, 0.99) |  |
| **All-cause mortality (n = 7,341)** | | |  |  |  |  |
| 3 months |  |  |  |  |  |  |
| Continuation | 6 | 15.97 | Ref. | 0.02 | Ref. | 0.40 |
| Cessation | 6 | 4.12 | 0.26 (0.08, 0.80) |  | 0.53 (0.12, 2.30) |  |
| 6 months |  |  |  |  |  |  |
| Continuation | 8 | 10.66 | Ref. | 0.03 | Ref. | 0.70 |
| Cessation | 11 | 3.77 | 0.35 (0.14, 0.88) |  | 0.79 (0.25, 2.57) |  |
| 12 months |  |  |  |  |  |  |
| Continuation | 28 | 18.74 | Ref. | <0.01 | Ref. | 0.26 |
| Cessation | 45 | 7.73 | 0.41 (0.26, 0.66) |  | 0.71 (0.39, 1.29) |  |
| 48 months |  |  |  |  |  |  |
| Continuation | 175 | 28.39 | Ref. | <0.01 | Ref. | 0.18 |
| Cessation | 441 | 17.87 | 0.63 (0.52, 0.74) |  | 0.86 (0.69, 1.07) |  |

*A total of 125 participants who experienced major bleeding prior to 13 June 2017 were removed from the major bleeding analysis. Abbreviation: CVD, cardiovascular disease; MACE, major adverse cardiovascular events; PS, propensity score; HR, hazard ratio; CI, confidence interval. **Possible aspirin indication defined as self-report of angina, heart/chest pain, transient ischemic attack, atrial fibrillation, deep vein thrombosis/ pulmonary embolism, any cardiac/vascular hospitalization; self-report of medications commonly used for secondary prevention (nitrates, ranolazine, or platelet aggregation inhibitors excluding aspirin); or any event sent to the MI, heart failure, or stroke adjudication committee but rejected as not meeting the outcome criteria.

|  | **Events** | **Incidence Rate (per 1000py)** | **Unadjusted HR**  **(95% CI)** | **p** | **PS-adjusted HR (95% CI)** | **p** |
| --- | --- | --- | --- | --- | --- | --- |
| **CVD (n = 5,637)** | |  |  |  |  |  |
| 3 months |  |  |  |  |  |  |
| Continuation | 2 | 13.71 | Ref. | 0.71 | Ref. | 0.77 |
| Cessation | 13 | 10.30 | 0.75 (0.17, 3.33) |  | 0.80 (0.17, 3.67) |  |
| 6 months |  |  |  |  |  |  |
| Continuation | 3 | 10.31 | Ref. | 1.00 | Ref. | 0.87 |
| Cessation | 26 | 10.32 | 1.00 (0.30, 3.31) |  | 1.11 (0.33, 3.76) |  |
| 12 months |  |  |  |  |  |  |
| Continuation | 8 | 13.79 | Ref. | 0.26 | Ref. | 0.26 |
| Cessation | 45 | 8.95 | 0.65 (0.31, 1.38) |  | 0.64 (0.30, 1.38) |  |
| 48 months |  |  |  |  |  |  |
| Continuation | 40 | 18.40 | Ref. | 0.03 | Ref. | 0.05 |
| Cessation | 262 | 12.93 | 0.70 (0.50, 0.97) |  | 0.72 (0.51, 1.01) |  |
| **MACE (n = 5,637)** | |  |  |  |  |  |
| 3 months |  |  |  |  |  |  |
| Continuation | 2 | 13.71 | Ref. | 0.56 | Ref. | 0.63 |
| Cessation | 11 | 8.72 | 0.64 (0.14, 2.87) |  | 0.68 (0.15, 3.21) |  |
| 6 months |  |  |  |  |  |  |
| Continuation | 3 | 10.31 | Ref. | 0.84 | Ref. | 0.99 |
| Cessation | 23 | 9.12 | 0.89 (0.27, 2.95) |  | 1.01 (0.29, 3.46) |  |
| 12 months |  |  |  |  |  |  |
| Continuation | 8 | 13.79 | Ref. | 0.17 | Ref. | 0.19 |
| Cessation | 41 | 8.15 | 0.59 (0.28, 1.26) |  | 0.59 (0.27, 1.29) |  |
| 48 months |  |  |  |  |  |  |
| Continuation | 27 | 12.39 | Ref. | 0.32 | Ref. | 0.30 |
| Cessation | 206 | 10.15 | 0.82 (0.55, 1.22) |  | 0.81 (0.53, 1.21) |  |
| **Major bleeding* (n = 5,571)** | | |  |  |  |  |
| 3 months |  |  |  |  |  |  |
| Continuation | 3 | 21.21 | Ref. | 0.01 | Ref. | 0.02 |
| Cessation | 4 | 3.20 | 0.15 (0.03, 0.68) |  | 0.16 (0.03, 0.76) |  |
| 6 months |  |  |  |  |  |  |
| Continuation | 3 | 10.61 | Ref. | 0.08 | Ref. | 0.15 |
| Cessation | 8 | 3.20 | 0.30 (0.08, 1.14) |  | 0.36 (0.09, 1.45) |  |
| 12 months |  |  |  |  |  |  |
| Continuation | 6 | 10.63 | Ref. | 0.02 | Ref. | 0.04 |
| Cessation | 17 | 3.40 | 0.32 (0.13, 0.81) |  | 0.36 (0.14, 0.96) |  |
| 48 months |  |  |  |  |  |  |
| Continuation | 24 | 11.37 | Ref. | <0.01 | Ref. | 0.01 |
| Cessation | 119 | 5.87 | 0.51 (0.33, 0.79) |  | 0.55 (0.35, 0.87) |  |
| **All-cause mortality (n = 5,637)** | | |  |  |  |  |
| 3 months |  |  |  |  |  |  |
| Continuation | 2 | 13.72 | Ref. | 0.09 | Ref. | 0.10 |
| Cessation | 4 | 3.17 | 0.23 (0.04, 1.26) |  | 0.23 (0.04, 1.32) |  |
| 6 months |  |  |  |  |  |  |
| Continuation | 2 | 6.87 | Ref. | 0.33 | Ref. | 0.72 |
| Cessation | 8 | 3.17 | 0.46 (0.10, 2.17) |  | 0.74 (0.15, 3.77) |  |
| 12 months |  |  |  |  |  |  |
| Continuation | 11 | 18.99 | Ref. | <0.01 | Ref. | 0.01 |
| Cessation | 33 | 6.55 | 0.34 (0.17, 0.68) |  | 0.41 (0.20, 0.84) |  |
| 48 months |  |  |  |  |  |  |
| Continuation | 58 | 24.42 | Ref. | 0.01 | Ref. | 0.09 |
| Cessation | 362 | 16.91 | 0.69 (0.52, 0.91) |  | 0.78 (0.59, 1.04) |  |

**Table S3.** The hazard of each study outcome in the aspirin cessation versus continuation group during four follow-up periods (additionally excluding participants with reported antithrombotic use).

*A total of 66 participants who experienced major bleeding prior to 13 June 2017 were removed from the major bleeding analysis. Abbreviation: CVD, cardiovascular disease; MACE, major adverse cardiovascular events; PS, propensity score; HR, hazard ratio; CI, confidence interval.

**Table S4.** Participant characteristics at *Time zero* (13 June 2017), with additional variables included and new ASDs calculated using different propensity scores.

|  | **Overall**  **(n=6,103)** | **Aspirin**  **Continuation**  **(n=676)** | **Aspirin**  **Cessation**  **(n=5,427)** | **Absolute standardized difference (ASD)** | |
| --- | --- | --- | --- | --- | --- |
|  |  |  |  | **Before** | **After** |
| Age, years (min-max) | 79.2 ± 4.4  (67.9-100.2) | 80.0 ± 4.8  (68.0-97.1) | 79.1 ± 4.3  (67.9-100.2) | 0.21 | 0.05 |
| Male gender | 2661 (43.6) | 292 (43.2) | 2369 (43.7) | <0.01 | <0.01 |
| Race/ethnicity/country |  |  |  |  |  |
| White Australian | 5293 (86.7) | 504 (74.6) | 4789 (88.2) | 0.14 | <0.01 |
| White US | 339 (5.6) | 72 (10.7) | 267 (4.9) | 0.06 | <0.01 |
| Hispanic | 253 (4.1) | 59 (8.7) | 194 (3.6) | 0.05 | <0.01 |
| Black | 135 (2.2) | 28 (4.1) | 107 (2.0) | 0.02 | <0.01 |
| Other | 83 (1.4) | 13 (1.9) | 70 (1.3) | <0.01 | <0.01 |
| Education ≥ 12 years | 3448 (56.5) | 414 (61.2) | 3034 (55.9) | 0.05 | <0.01 |
| BMI, kg/m^2*^ | 27.7 (4.7) | 28.2 (4.8) | 27.6 (4.7) | 0.12 | 0.03 |
| Total cholesterol, mg/DL | 197.2 ± 38.8 | 188.3 ± 41.6 | 198.3 ± 38.3 | 0.26 | 0.02 |
| Triglycerides, mg/DL | 118.5 ± 56.6 | 117.7 ± 53.6 | 118.6 ± 56.9 | 0.02 | 0.03 |
| HDL-c, mg/DL* | 61.9 (17.6) | 60.9 (17.0) | 62.1 (17.7) | 0.07 | 0.02 |
| Fasting plasma glucose, mg/DL* | 99.6 (20.4) | 102.9 (31.4) | 99.2 (18.6) | 0.19 | 0.01 |
| SBP, mmHg | 136.9 ± 16.9 | 137.1 ± 17.3 | 136.9 ± 16.8 | 0.01 | 0.01 |
| DBP, mmHg | 74.2 ± 9.7 | 74.0 ± 9.8 | 74.2 ± 9.7 | 0.02 | 0.01 |
| Smoking, ever | 2621 (42.9) | 302 (44.7) | 2319 (42.7) | 0.02 | <0.01 |
| Alcohol, ever* | 5288 (86.6) | 568 (84.0) | 4720 (87.0) | 0.03 | <0.01 |
| eGFR, ml/min/1.73m^2^ | 69.9 ± 14.4 | 68.8 ± 15.4 | 70.0 ± 14.2 | 0.09 | <0.01 |
| Self-report diabetes | 600 (9.8) | 98 (14.5) | 502 (9.3) | 0.05 | <0.01 |
| Statin use | 2206 (36.1) | 341 (50.4) | 1865 (34.4) | 0.16 | 0.02 |
| Anti-hypertensive agents use | 3630 (59.5) | 475 (70.3) | 3155 (58.1) | 0.12 | 0.02 |
| NSAID use | 901 (14.8) | 117 (17.3) | 784 (14.4) | 0.03 | <0.01 |
| Anti-thrombotic use (excluding aspirin) | 466 (7.6) | 92 (13.6) | 374 (6.9) | 0.07 | <0.01 |
| Family History of MI | 2571 (42.1) | 320 (47.3) | 2251 (41.5) | 0.06 | <0.01 |
| Grip strength (kg)* | 25.6 (9.4) | 24.8 (9.9) | 25.6 (9.4) | 0.09 | <0.01 |
| Gait speed (m/sec)* | 1.0 (0.2) | 0.9 (.2) | 1.0 (0.2) | 0.31 | 0.06 |
| Pre-trial aspirin use (*not adjusted*) | 675 (11.1) | 134 (19.8) | 541 (10.0) | -- | -- |

Abbreviation: SBP, systolic blood pressure; DBP, diastolic blood pressure; eGFR, estimated glomerular filtration rate; MI, myocardial infarction; NSAIDs, Non-steroidal anti-inflammatory drugs.

Continuous variables are presented as mean ± SD and categorical variables are presented as n (%).

*Variables included in the propensity score only in sensitivity analyses.

**Table S5.** The hazard of each study outcome in the aspirin cessation group versus the continuation group during four follow-up periods (with additional covariates in propensity score model: HDL-c, fasting plasma glucose, alcohol consumption, BMI, grip strength, gait speed).

|  | **Events** | **Incidence Rate (per 1000py)** | **Unadjusted HR**  **(95% CI)** | **p** | **PS-adjusted HR (95% CI)** | **p** |
| --- | --- | --- | --- | --- | --- | --- |
| **CVD (n = 6,103)** | |  |  |  |  |  |
| 3 months |  |  |  |  |  |  |
| Continuation | 2 | 11.85 | Ref. | 1.0 | Ref. | 0.72 |
| Cessation | 16 | 11.81 | 1.00 (0.23, 4.34) |  | 1.32 (0.29, 6.10) |  |
| 6 months |  |  |  |  |  |  |
| Continuation | 3 | 8.90 | Ref. | 0.72 | Ref. | 0.46 |
| Cessation | 30 | 11.09 | 1.25 (0.38, 4.08) |  | 1.59 (0.47, 5.42) |  |
| 12 months |  |  |  |  |  |  |
| Continuation | 9 | 13.39 | Ref. | 0.28 | Ref. | 0.40 |
| Cessation | 49 | 9.07 | 0.68 (0.33, 1.38) |  | 0.73 (0.35, 1.52) |  |
| 48 months |  |  |  |  |  |  |
| Continuation | 46 | 18.20 | Ref. | 0.03 | Ref. | 0.13 |
| Cessation | 281 | 12.94 | 0.71 (0.52, 0.96) |  | 0.78 (0.56, 1.08) |  |
| **MACE (n = 6,103)** | |  |  |  |  |  |
| 3 months |  |  |  |  |  |  |
| Continuation | 2 | 11.85 | Ref. | 0.86 | Ref. | 0.83 |
| Cessation | 14 | 10.33 | 0.87 (0.20, 3.84) |  | 1.19 (0.25, 5.58) |  |
| 6 months |  |  |  |  |  |  |
| Continuation | 3 | 8.90 | Ref. | 0.85 | Ref. | 0.53 |
| Cessation | 27 | 9.98 | 1.12 (0.34, 3.70) |  | 1.48 (0.43, 5.10) |  |
| 12 months |  |  |  |  |  |  |
| Continuation | 8 | 11.90 | Ref. | 0.35 | Ref. | 0.50 |
| Cessation | 45 | 8.33 | 0.70 (0.33, 1.48) |  | 0.77 (0.35, 1.67) |  |
| 48 months |  |  |  |  |  |  |
| Continuation | 31 | 12.22 | Ref. | 0.33 | Ref. | 0.54 |
| Cessation | 221 | 10.16 | 0.83 (0.57, 1.21) |  | 0.88 (0.60, 1.30) |  |
| **Major bleeding* (n = 6,028)** | | |  |  |  |  |
| 3 months |  |  |  |  |  |  |
| Continuation | 3 | 18.35 | Ref. | 0.02 | Ref. | 0.03 |
| Cessation | 4 | 2.98 | 0.16 (0.04, 0.73) |  | 0.17 (0.04, 0.80) |  |
| 6 months |  |  |  |  |  |  |
| Continuation | 3 | 9.18 | Ref. | 0.10 | Ref. | 0.17 |
| Cessation | 8 | 2.98 | 0.32 (0.09, 1.22) |  | 0.37 (0.09, 1.51) |  |
| 12 months |  |  |  |  |  |  |
| Continuation | 6 | 9.20 | Ref. | 0.02 | Ref. | 0.04 |
| Cessation | 17 | 3.17 | 0.34 (0.14, 0.87) |  | 0.36 (0.14, 0.95) |  |
| 48 months |  |  |  |  |  |  |
| Continuation | 26 | 10.59 | Ref. | 0.01 | Ref. | 0.06 |
| Cessation | 136 | 6.27 | 0.58 (0.38, 0.89) |  | 0.66 (0.42, 1.01) |  |
| **All-cause mortality (n = 6,103)** | | |  |  |  |  |
| 3 months |  |  |  |  |  |  |
| Continuation | 2 | 11.85 | Ref. | 0.11 | Ref. | 0.09 |
| Cessation | 4 | 2.95 | 0.25 (0.05, 1.36) |  | 0.22 (0.04, 1.25) |  |
| 6 months |  |  |  |  |  |  |
| Continuation | 2 | 5.93 | Ref. | 0.46 | Ref. | 0.69 |
| Cessation | 9 | 3.32 | 0.56 (0.12, 2.59) |  | 0.72 (0.14, 3.60) |  |
| 12 months |  |  |  |  |  |  |
| Continuation | 12 | 17.88 | Ref. | <0.01 | Ref. | 0.01 |
| Cessation | 35 | 6.47 | 0.36 (0.19, 0.70) |  | 0.40 (0.20, 0.80) |  |
| 48 months |  |  |  |  |  |  |
| Continuation | 69 | 25.06 | Ref. | <0.01 | Ref. | 0.20 |
| Cessation | 395 | 17.18 | 0.68 (0.53, 0.88) |  | 0.84 (0.64, 1.10) |  |

*A total of 75 participants who experienced major bleeding prior to 13 June 2017 were removed from the major bleeding analysis. Abbreviation: CVD, cardiovascular disease; BMI, body mass index; HDL-c, high-density-lipoprotein cholesterol; MACE, major adverse cardiovascular events; PS, propensity score; HR, hazard ratio; CI, confidence interval.
